# Identification of SDC4 as a potential target for obesity via integrated analysis of the lncRNA-miRNA-mRNA network in visceral adipose tissue

**DOI:** 10.1080/21623945.2025.2583542

**Published:** 2025-11-06

**Authors:** Yuancheng Shao, Feng Ju, Jun Qian, Liming Tang, Shuai Chen

**Affiliations:** Center of Gastrointestinal Disease, The Affiliated Changzhou NO. 2 People’s Hospital of Nanjing Medical University, Changzhou, China

**Keywords:** adipocyte, LncRNA, miRNA, ceRNA, SDC4

## Abstract

Obesity is a major global health issue. This study aimed to elucidate its molecular mechanisms by analysing the expression of lncRNAs, miRNAs, and mRNAs in visceral adipose tissue. Through integrated transcriptome sequencing and bioinformatics analysis of obese and normal groups, we observed 118, 92 and 227 differentially expressed lncRNAs, miRNAs and mRNAs, respectively. Functional enrichment analysis revealed these genes were primarily involved in immune response and inflammation-related pathways. A competing endogenous RNA (ceRNA) network was constructed, identifying key interactions among five target genes, including SDC4. Validation confirmed SDC4 was significantly upregulated in obese subjects, and this expression level positively correlated with body mass index and triglyceride. These findings suggest that SDC4 offers the possibility of being a therapeutic target for obesity.

## Introduction

The global prevalence of obesity has surged dramatically in recent decades, establishing it as one of the most pressing public health crises of the modern era [1]. Obesity is recognized as a complex, multisystem disease intricately linked to numerous comorbidities that significantly elevate morbidity and mortality rates [2]. Beyond its direct health impacts, the obesity epidemic imposes a substantial socioeconomic burden, contributing to unemployment, social disadvantage, and diminished economic productivity [3]. Effectively mitigating this escalating burden necessitates the implementation of comprehensive, multi-tiered preventive strategies and public health policies.

A primary hallmark of obesity is the excessive accumulation of adipose tissue, which not only leads to weight gain but also precipitates a cascade of metabolic disturbances, including decreased insulin sensitivity and chronic inflammation. The pathological expansion of adipose tissue in obesity triggers adipocyte death, hypoxia, and mechanical stress, which collectively initiate a state of chronic low-grade inflammation within the tissue [4]. This inflammatory milieu is not confined to the adipose depot; it exerts systemic effects by releasing pro-inflammatory cytokines that disrupt insulin signalling pathways in peripheral tissues [5,6]. Furthermore, obesity is characterized by the ectopic deposition of lipids in non-adipose organs such as the liver, skeletal muscle, and pancrea, a process widely recognized as a key aetiological factor in the development of metabolic syndrome [7,8]. The accumulation of lipids in these organs induces lipotoxicity, impairing their normal metabolic functions and further exacerbating systemic insulin resistance [9]. Therefore, elucidating the molecular mechanisms governing adipose tissue remodelling is of paramount importance for developing novel therapeutic and preventive strategies against obesity-related metabolic complications.

The competing endogenous RNA (ceRNA) regulatory network represents a crucial layer of gene expression control [10]. Within this network, long non-coding RNAs (lncRNAs) have been identified as essential regulators of adipocyte physiology [11,12]. By acting as molecular sponges for microRNAs (miRNAs), lncRNAs can competitively bind to and sequester these miRNAs, thereby modulating the expression of target genes and influencing adipocyte physiology [13]. The involvement of lncRNAs in obesity extends beyond lipid metabolism to regulate inflammatory responses and signalling transduction. Given that obesity is often accompanied by chronic low-grade inflammation, lncRNAs have been shown to influence immune activation and the development, differentiation, and mobilization of T cells by regulating the production of inflammatory mediators and cell migration [14,15]. Beyond their roles as ceRNAs, some lncRNAs function as molecular scaffolds or guides, engaging in sophisticated crosstalk with proteins, RNAs, and genomic DNA [16,17]. Through these versatile mechanisms, they are implicated in multiple aspects of obesity’s pathophysiology.

To elucidate the molecular mechanisms of obesity and identify novel therapeutic targets, we conducted a comprehensive analysis of visceral adipose tissue (VAT) using whole-transcriptome sequencing coupled with bioinformatics approaches, including differential expression, GO, and KEGG pathway enrichment analyses. This strategy enabled the systematic characterization of RNA expression changes and was extended by the construction of a ceRNA regulatory network to further decipher the complex interplay and functional implications of various RNAs in obesity-related pathophysiology.

## Materials and methods

### Whole-transcriptome sequencing

Total RNA was extracted from adipose tissue samples, and ribosomal RNA (rRNA) was subsequently depleted using the Ribo-Zero Gold rRNA Removal Kit (Illumina, USA) following the manufacturer’s instructions. All the samples were processed in the same batch and there was no batch effect.

The purified RNA was fragmented into short segments of approximately 200–300 base pairs (bp). First-strand complementary DNA (cDNA) was synthesized using random hexamer primers. For second-strand cDNA synthesis, dUTP was incorporated instead of dTTP to mark the second strand. After adapter ligation, the strand-specificity was achieved by digesting the dUTP-containing second strand with Uracil-N-Glycosylase (UNG), thereby ensuring that only the first strand was preserved. The resulting single-stranded cDNA was then subjected to end-repair, 3’dA-tailing, and sequencing adapter ligation. The final library was constructed after size selection and polymerase chain reaction (PCR) amplification.

The quality and size distribution of the constructed libraries were assessed using an Agilent 2100 Bioanalyzer (Agilent Technologies, USA). Libraries that passed quality control were subsequently subjected to high-throughput sequencing on an Illumina NovaSeq 6000 platform (Illumina, USA) to generate 150 bp paired-end reads.

Based on the characteristics of lncRNAs, four software programs, namely CPC [18], CNCI [19], Pfam [20], and PLEK [21], were used to obtain candidate lncRNAs through a strict four-step screening method: The first step: compare and analyse the spliced transcripts with the reference transcripts (reference_transcripts) to screen out new transcripts with known coding or known loci. In this step, the cuffcompare software is used to compare merged_transcripts with reference_transcripts one by one to determine the location types of the remaining transcripts. Then, through the screening of candidate lncRNA transcripts, those marked with ‘i’, ‘u’, ‘x’, or ‘o’ are retained and proceed to the second screening step. Step 2: Screen the transcripts obtained from the first step based on a length greater than 200nt and an exon number of 2 or more. Step 3: Use CPC, CNCI, Pfam, and PLEK software to predict and analyse the coding ability of the transcripts screened in Step 2, and screen out the transcripts with coding potential. Step 4: For species with known lncRNAs, the lncRNA sequences predicted in Step 3 are compared with the known lncRNAs using blastn software. Those identified in the comparison are recognized as known lncRNAs. After merging with known lncRNA sequences, quantitative analysis was conducted. For species without known lncRNAs, the lncRNA sequences predicted in the third step are directly used for quantitative analysis.

The raw sequencing data generated in this study have been deposited in the Gene Expression Omnibus (GEO) database at the National Center for Biotechnology Information (NCBI) and are accessible through the GEO Series accession number GSE235696. The expression matrix can be obtained from GSE235696. Adipose tissue samples were obtained from patients undergoing laparoscopic hernia repair and bariatric surgery, and written informed consent was obtained from all participants. Population-related information is provided in Table 1. This study was conducted in accordance with the ethical principles of the Declaration of Helsinki and was approved by the Ethics Committee of The Affiliated Changzhou No. 2 People’s Hospital of Nanjing Medical University (Approval No. 2021YLA001). The methods employed were consistent with those outlined in our previous study [22].

**Table 1.** Visceral adipose tissue received whole transcriptome sequencing population information.

Samples	Age (year)	Sex	BMI (Kg/m^2^)	TC (mmol/L)	TG (mmol/L)	HDL-C (mmol/L)	LDL-C (mmol/L)
Normal 1	33	Famale	24.03	3.92	0.72	1.47	2.13
Normal 2	30	Male	23.26	3.76	0.93	1.42	2.05
Normal 3	24	Male	20.75	3.65	1.05	1.86	1.53
Normal 4	35	Male	23.88	4.47	1.17	1.32	2.6
Obese 1	33	Famale	40.19	6.36	2.2	1.19	4.26
Obese 2	29	Famale	38.30	4.99	3.48	1.25	3.09
Obese 3	24	Male	39.65	5.56	4.32	0.65	3.42
Obese 4	37	Male	39.70	6.61	1.17	1.17	4.51

### Differential expression analysis

To identify differentially expressed lncRNAs (DElncRNAs), miRNAs (DEmiRNAs), and mRNAs (DEmRNAs), we conducted an analysis using the R software packages DESeq2 [23]. For lncRNAs and mRNAs, transcripts were considered differentially expressed if they met the criteria of an absolute log_2_ (fold change) (|log_2_FC|) greater than 1 and an adjusted *p* value (q-value) below 0.05. A distinct set of thresholds was applied for miRNAs, where significance was defined as |log_2_FC| > 0.58 (equivalent to a 1.5-fold change) and an unadjusted *p* value < 0.05.

### GO and KEGG pathway enrichment analysis

In order to clarify the biological roles of the DEmRNAs, GO and KEGG pathway enrichment analyses were performed utilizing the clusterProfiler R package. GO and KEGG pathway enrichment analyses were performed exclusively on the list of differentially expressed protein-coding mRNAs. An unadjusted *p* value threshold of less than 0.05 was established to denote statistical significance.

### Construction of the ceRNA regulatory network

The initial step in constructing the ceRNA regulatory network involved predicting the potential targeting relationships between DElncRNAs and DEmiRNAs with the aid of the miRanda software [24]. Subsequently, to enhance the prediction accuracy, we identified potential targeting sites between DEmiRNAs and DEmRNAs by integrating the results from three databases: miRDB [25], miRWalk [26], and TargetScan [27]. The intersection of predictions from these three databases was taken as the final set of miRNA-mRNA interactions.

Based on the identified lncRNA-miRNA and miRNA-mRNA targeting relationships, an integrated lncRNA-miRNA-mRNA ceRNA regulatory network was constructed. Finally, the constructed ceRNA network was visualized using Cytoscape software (v3.9.1).

### Quantitative real-time PCR (RT-qPCR) validation

To validate the experiments, total RNA was isolated and subsequently converted into cDNA through reverse transcription. This was followed by RT-qPCR employing SYBR Green to evaluate the expression levels of the target genes. The relative expression levels were determined utilizing the 2^(-ΔΔCt) method, normalizing against a housekeeping gene. The specific sequences of the primers employed in this research are detailed in Table 2.

**Table 2.** Primer sequences.

Gene	Sequences (5’-3’)
GAPDH F	TGCAACCGGGAAGGAAATGA
GAPDH R	GCATCACCCGGAGGAGAAAT
CYP1A2 F	CTTCGGACAGCACTTCCCTG
CYP1A2 R	AGGGTTAGGCAGGTAGCGA
TNFAIP3 F	CCGCCAAGAGAGATCACACC
TNFAIP3 R	TTCGCAAAGTCCCAAGTCCT
EREG F	ACCCTGGGGAGTCTATGGTC
EREG R	GTCGTGAGTTGGCATAGGGA
ITPKC F	CACGTCATCCTGGTGGTAGG
ITPKC R	CACCGTCTTGCCGAAGTCTA
SDC4 F	GAGCCCTACCAGACGATGAG
SDC4 R	GGCCGATCATGGAGTCTTCC

### Statistical analysis

Statistical evaluations and graphical illustrations were performed utilizing GraphPad Prism 8.0 and R software. For comparisons between two groups, a two-tailed unpaired Student’s t-test was used. The relationship between variables was evaluated through Spearman’s rank correlation analysis, which determined both the strength and direction of associations. Clinical variables were compared between low and high SDC4 expression groups to assess potential associations. For continuous variables [body weight, body mass index (BMI), HbA1c, alanine aminotransferase (ALT), aspartate aminotransferase (AST), total Cholesterol (TC), triglyceride (TG), high density lipoprotein cholesterol (HDL-C) and low density lipoprotein cholesterol (LDL-C)], normality was assessed using the Shapiro-Wilk test. Student’s t-test was applied for normally distributed data, while the Mann-Whitney U test was used for non-normally distributed data. For categorical variables Hypertension, Type II Diabetes, Hyperuricaemia, non-alcoholic fatty liver disease (NAFLD), and Cholelithiasis), chi-square test or Fisher’s exact test was performed based on expected cell frequencies (Fisher’s exact test was used when any expected cell frequency was < 5). All quantitative data are expressed as the mean ± standard deviation (mean ± SD), with a *p* value threshold of less than 0.05 established to indicate statistical significance.

## Results

### Identification of DElncRNAs, DEmiRNAs and DEmRNAs in vat

**Figure 1.**
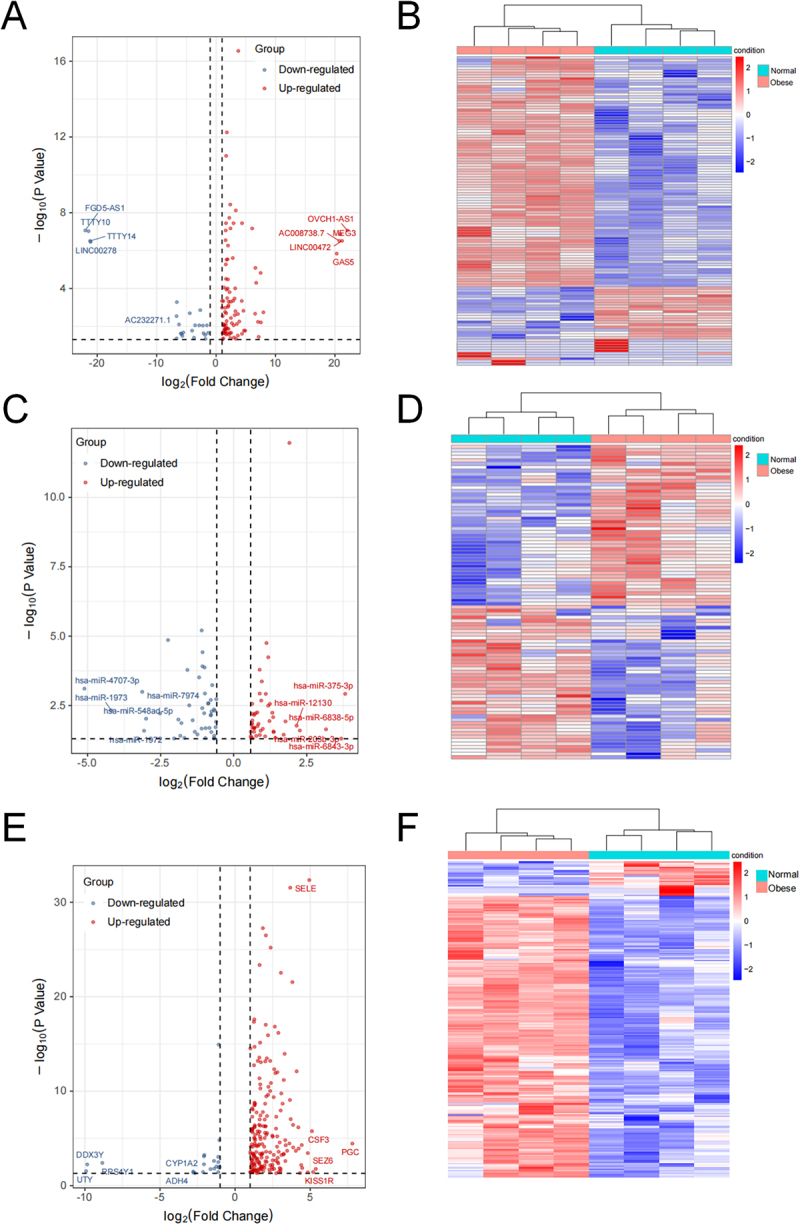
Differential expression analysis of lncRnas, miRnas, and mRNAs in visceral adipose tissue (a) Volcano plot depicting differentially expressed lncRnas (DElncRnas). Red dots represent upregulated lncRnas (*n* = 93) and blue dots represent downregulated lncRnas (*n* = 25). (b) Heatmap illustrating hierarchical clustering of DElncRNAs. Rows represent individual samples, and columns represent lncRnas. Color intensity indicates expression level (red: high expression; blue: low expression). (c) Volcano plot depicting differentially expressed miRnas (DEmiRnas). Red dots represent upregulated miRnas (*n* = 47) and blue dots represent downregulated miRnas (*n* = 45). (d) Heatmap illustrating hierarchical clustering of DE-miRnas. (e) Volcano plot depicting differentially expressed mRNAs (DEmRNAs). Red dots represent upregulated mRNAs (*n* = 202) and blue dots represent downregulated mRNAs (*n* = 25). (f) heatmap illustrating hierarchical clustering of DEmRNAs.

To explore the potential roles of non-coding RNAs and mRNAs in obesity, we performed whole-transcriptome sequencing on VAT samples from 4 normal healthy individuals (normal group) and 4 obese individuals (obese group). Differential expression analysis was conducted for lncRNAs, miRNAs, and mRNAs, respectively. Clustering of samples by expression profiles showed a clear separation of obese vs normal VAT (Supplementary figure S1), indicating distinct transcriptomic patterns. We identified numerous differentially expressed genes between the groups, as detailed below. A comparative analysis between the obese and normal groups revealed significant alterations in non-coding RNA and mRNA expression profiles. Specifically, we identified 118 DElncRNAs, comprising 93 upregulated and 25 downregulated transcripts in the obese group (Figure 1(A,B)). Among the DElncRNAs between obese and normal VAT, the top five upregulated were OVCH1-AS1, LINC00472, MEG3, AC008738.7, and GAS5 (Supplementary table S1). Conversely, the top five downregulated were FGD5-AS1, TTTY10, TTTY14, LINC00278, and AC232271.1 (Supplementary table S1). Similarly, 92 DEmiRNAs were detected, with 47 showing increased and 45 showing decreased expression (Figure 1(C,D)). A comparison of obese and normal VAT revealed the top five upregulated miRNAs: hsa-miR-375-3p, hsa-miR-6843-3p, hsa-miR-6838-5p, hsa-miR-203b-3p, and hsa-miR-12130, and the most significantly downregulated miRNAs were hsa-miR-4707-3p, hsa-miR-1973, hsa-miR-7974, hsa-miR-1972, and hsa-miR-548ad-5p (Supplementary table S2). Furthermore, the analysis uncovered 227 DEmRNAs, of which 202 were upregulated and 25 were downregulated in the obese group relative to the normal group (Figure 1(E,F)). In the differential expression analysis of VAT from obese versus normal subjects, the five genes with the highest upregulation were PGC, SEZ6, KISS1R, CSF3 and SELE (Supplementary table S3). In contrast, the five most downregulated genes were UTY, DDX3Y, RPS4Y1, ADH4, and CYP1A2 (Supplementary table S3). These results indicate significant alterations in the expression profiles of LncRNAs, miRNAs, and mRNAs in the VAT of obese individuals, suggesting their potential involvement in the pathogenesis of obesity.

### GO and KEGG pathway enrichment analyses of DEmRNAs

**Figure 2.**
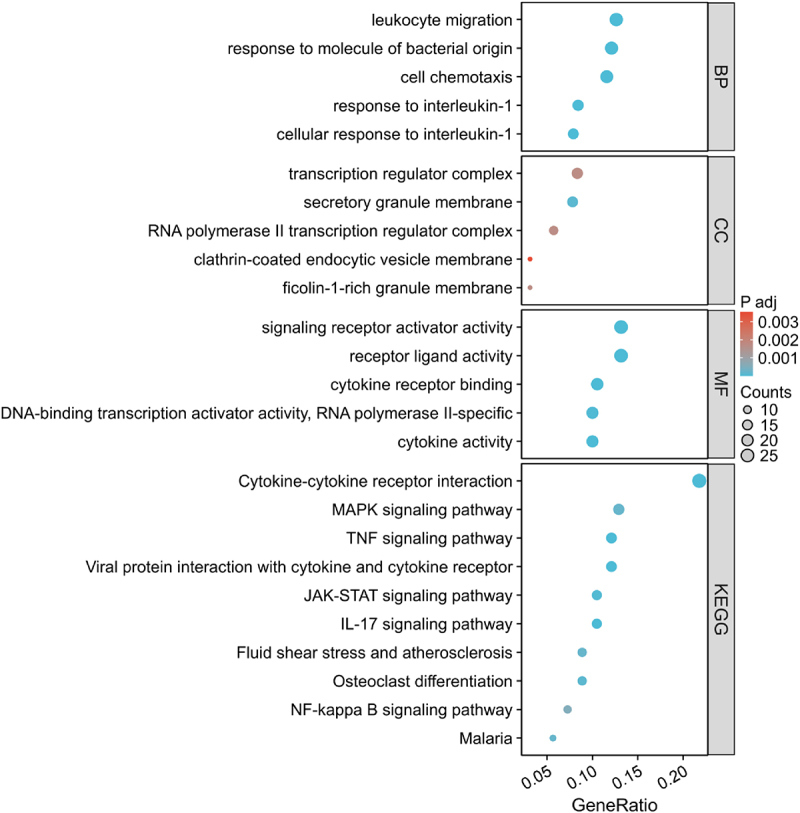
Gene ontology (go) and KEGG pathway enrichment analysis of differentially expressed mRNAs dot plots displaying the enrichment results of go terms and KEGG pathways for differentially expressed mRNAs. For biological process (bp), cellular component (cc), and molecular function (mf) categories, the top 5 enriched terms are shown. For KEGG pathways, the top 10 enriched pathways are displayed.

To further elucidate the underlying biological mechanisms between the two adipose tissue groups, GO and KEGG pathway enrichment analyses were performed on the DEmRNAs (Figure 2 and Supplementary table S4).

The GO enrichment analysis revealed that the top five enriched biological processes (BP) included leukocyte migration, response to molecule of bacterial origin, cell chemotaxis, response to interleukin-1, and cellular response to interleukin-1. These findings suggest that the DEmRNAs are predominantly involved in immune responses, inflammatory reactions, and cell migration processes.

In terms of cellular components (CC), the most significantly enriched terms were transcription regulator complex, secretory granule membrane, RNA polymerase II transcription regulator complex, clathrin-coated endocytic vesicle membrane, and ficolin-1-rich granule membrane, indicating that these genes play crucial roles in transcriptional regulation, vesicular transport, and secretory functions.

For molecular functions (MF), the enriched terms included signalling receptor activator activity, receptor ligand activity, cytokine receptor binding, DNA-binding transcription activator activity, RNA polymerase II-specific, and cytokine activity. These results highlight the key roles of these genes in signal transduction, transcriptional regulation, and cytokine-mediated immune modulation.

The KEGG pathway analysis identified the top 10 significantly enriched pathways, including Cytokine-cytokine receptor interaction, MAPK signalling pathway, TNF signalling pathway, Viral protein interaction with cytokine and cytokine receptor, JAK-STAT signalling pathway, IL-17 signalling pathway, Fluid shear stress and atherosclerosis, Osteoclast differentiation, NF-kappa B signalling pathway, and Malaria. These pathways are primarily associated with immune and inflammatory responses, such as cytokine-mediated signal transduction, inflammatory regulation, and disease-related processes.

Collectively, these enrichment analyses offer a valuable resource for understanding the role of DEmRNAs in immune regulation and the pathogenesis of obesity-related metabolic disorders.

### Construction of the lncRNA-miRNA-mRNA ceRNA regulatory network

**Figure 3.**
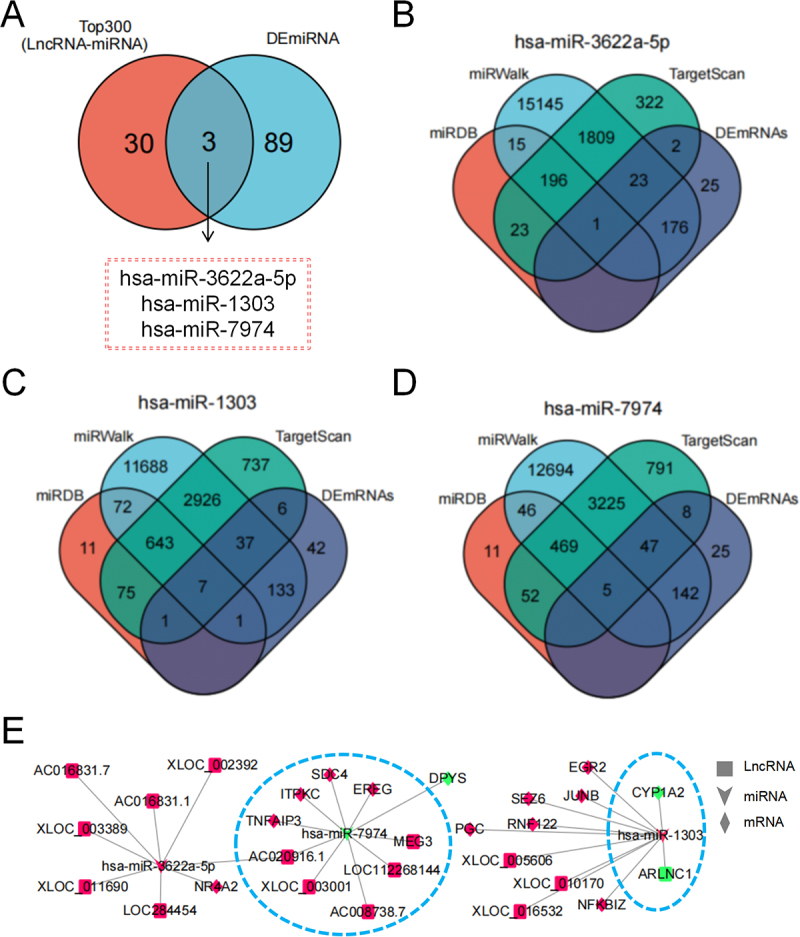
Construction of the lncRNA-miRNA-mRNA ceRNA regulatory network (a) Venn diagram showing the intersection between the top 300 predicted lncRNA-miRNA interactions and the differentially expressed miRnas (DEmiRnas). Three common miRnas (hsa-miR-3622a-5p, hsa-miR-1303, and hsa-miR-7974) were identified. (B-D) Venn diagrams depicting the overlap of predicted target mRNAs for each of the three selected miRnas (hsa-miR-3622a-5p in B, hsa-miR-1303 in C, and hsa-miR-7974 in d) Obtained from three different prediction algorithms (TargetScan, miRDB, and miRwalk) and the differentially expressed mRNAs (DEmRNAs). (e) Integrated ceRNA regulatory network diagram illustrating the interactions among lncRnas, miRnas, and mRNAs. Nodes represent molecules (lncRnas in squares, miRnas in V shapes, mRNAs in diamonds), and edges represent regulatory relationships. Red represents high expression in obese visceral adipose tissue and green represents low expression.

To explore the potential ceRNA regulatory mechanisms in VAT, we first predicted miRNAs that may interact with the DElncRNAs. By analysing the top 300 lncRNA-miRNA pairs based on prediction scores, we identified 33 candidate miRNAs (Figure 3(A)). Subsequently, we intersected these 33 miRNAs with the DEmiRNAs identified in our sequencing data. This analysis revealed three overlapping miRNAs: hsa-miR-3622a-5p, hsa-miR-1303, and hsa-miR-7974. To further construct the ceRNA network, we predicted the target mRNAs of these three miRNAs and intersected them with the DEmRNAs. The results indicated that: hsa-miR-3622a-5p potentially targets NR4A2; hsa-miR-1303 potentially targets EGR2, SEZ6, JUNB, RNF122, PGC, NFKBIZ, and CYP1A2; hsa-miR-7974 potentially targets DPYS, SDC4, ITPKC, EREG, and TNFAIP3 (Figure 3(B-D)). Then, a comprehensive lncRNA-miRNA-mRNA ceRNA network was constructed based on the identified upstream and downstream regulatory relationships, and the resulting network was visualized (Figure 3(E)). This network highlights several lncRNA-miRNA-mRNA interactions of interest for understanding the post-transcriptional regulatory mechanisms involved in obesity-related adipose tissue dysfunction.

### Validation of key gene expression and clinical correlation analysis

The ceRNA mechanism involves lncRNAs acting as molecular decoys for miRNAs. By competitively binding to miRNAs, lncRNAs shield target mRNAs from miRNA-mediated degradation or translational repression. According to the expression difference of lncRNA in VAT, it is consistent with mRNA and opposite to miRNA. Based on this regulatory mechanism, the highlighted ceRNA subnetwork was prioritized for further experimental validation (Figure 3(E)).

**Figure 4.**
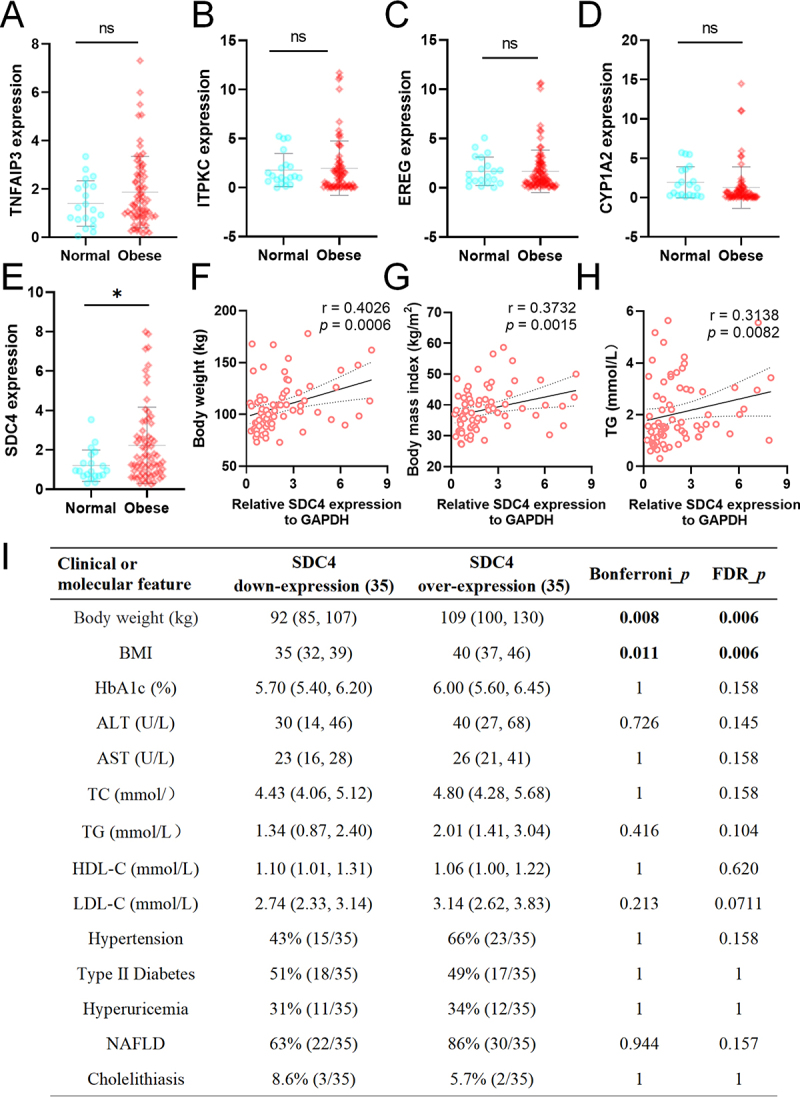
Validation of SDC4 expression and its association with obesity-related phenotypes (A- D) Expression levels of TNFAIP3 (a), ITPKC (b), EREG (c), and CYP1A2 (d) in visceral adipose tissues from normal (*n* = 20) and obese (*n* = 70) individuals. No significant differences were observed between the two groups (ns, *p* > 0.05). (e) SDC4 expression was significantly higher in obese individuals compared to normal individuals (*p < 0.05). (F-H) scatter plots showing positive correlations between SDC4 expression and body weight (f), body mass index (bmi) (g), and serum triglyceride (tg) levels (h) in the combined cohort (*r* = 0.4026, *p* = 0.0006; *r* = 0.3732, *p* = 0.00015; *r* = 0.3138, *p* = 0.0082, respectively). (i) Summary table presenting the association between SDC4 expression (divided into lower and upper tertiles) and various clinical or molecular features in the obese group (*n* = 35). Significant associations were found between SDC4 overexpression and increased body weight and BMI.

We subsequently validated the expression levels of five candidate genes (TNFAIP3, ITPKC, EREG, CYP1A2, and SDC4) in an expanded cohort consisting of VATs from 20 normal individuals and 70 obese individuals. While the expression levels of TNFAIP3, ITPKC, EREG, and CYP1A2 showed no significant differences between the obese and normal groups (Figure 4(A-D)), SDC4 was significantly upregulated in the VAT of obese individuals compared to normal individuals (Figure 4(E)).

Furthermore, SDC4 expression levels were positively correlated with body weight (*r* = 0.4026, *p* = 0.0006), BMI (*r* = 0.3732, *p* = 0.0015), and serum triglyceride levels (*r* = 0.3138, *p* = 0.0082, Figure 4(F,G)). The population was divided into two groups according to the expression level of SDC4. SDC4 expression was positively correlated with body weight and BMI (Figure 4(I)).

Finally, we verified the correlation between ceRNA networks targeting SDC4 within the sequencing data results. The results showed that MEG3, AC020916.1, AC008738.7, XLOC_003001 and LOC112268144 were negatively correlated with hsa-miR-7974 expression levels but without statistical significance. hsa-miR-7974 was also negatively correlated with SDC4, but there was no statistical significance. In addition, the expression levels of MEG3, AC008738.7 and XLOC_003001 were positively correlated with SDC4 (*r* = 0.83, *p* < 0.05; *r* = 0.21, *p* < 0.05; *r* = 0.71,*p* < 0.05. Supplementary figure S2).

## Discussion

The global obesity epidemic demands more effective therapies, yet current management strategies are limited. Lifestyle modifications suffer from poor long-term adherence; pharmacological agents are constrained by adverse effects and contraindications; and bariatric surgery, while most effective, is restricted by its invasiveness, cost, and limited eligibility [28–30]. These limitations underscore the need for novel therapeutic targets and more effective interventions.

In this study, we addressed this need by conducting a comprehensive transcriptomic analysis of VAT from obese and normal individuals. Through integrated bioinformatics analyses, we identified numerous lncRNAs, miRNAs, and mRNAs. By constructing a ceRNA network, we uncovered key regulatory interactions. The identification of a ceRNA network, where MEG3, AC020916.1, AC008738.7, XLOC_003001, and LOC112268144 act as molecular sponges for hsa-miR-7974 to regulate SDC4 expression, suggests potential new insights into the pathogenesis of obesity. The role of MEG3 in our findings is particularly compelling given its established involvement in adipose tissue biology. Previous studies have demonstrated that MEG3 acts as a ceRNA, sponging miR-21 to regulate LRP6 expression and influence hepatic lipogenesis [31], and modulating the miR-217/Dkk3 axis to promote preadipocyte differentiation [32]. Consistent with a role in adiposity, we observed significant upregulation of MEG3 in the VAT of obese patients, where it is positioned to regulate downstream genes within our ceRNA network. While some reports suggest that MEG3 knockdown promotes adipocyte differentiation [33], indicating potential context-dependent functions, our data solidify its association with human obesity. Similarly, the lncRNA AI504432 has been shown to regulate FASN expression by binding miR-1a-3p, thereby influencing lipogenesis in senescent adipocytes [34]. Collectively, these studies, alongside our own, reinforce the critical role of lncRNAs functioning as ceRNAs in the complex regulation of adipocyte metabolism.

MiRNAs, as another crucial component of the ceRNA hypothesis, are well-established post-transcriptional regulators of adipogenesis and adipocyte function [35,36]. For instance, miR-425 significantly impacts adipogenesis and lipolysis by targeting key signalling pathways such as PPARγ and AMPK [37], and miR-204-5p exerts a pro-adipogenic effect on human adipose-derived mesenchymal stem cells, a process mediated by its targeted repression of the Wnt/β-catenin pathway [38]. Our study identified hsa-miR-3622a-5p, hsa-miR-1303, and hsa-miR-7974 as central hubs within the obesity-associated ceRNA network. Notably, there is a paucity of research linking these specific miRNAs to adipose metabolism. This provides a clear direction for future investigation; functional validation of these miRNAs and their interactions within the network is an important next step to elucidate their precise roles in obesity pathophysiology.

To gain deeper insights into the potential biological functions and pathway associations of the aberrantly expressed genes, we employed GO and KEGG pathway enrichment analyses. Significant enrichment was observed in pathways central to immune response and inflammation. Among these, the cytokine-cytokine receptor interaction, MAPK signalling, and TNF signalling pathways were identified as the most prominently enriched. This suggests that chronic low-grade inflammation is a hallmark of the obese VAT microenvironment in our cohort. This finding aligns with established knowledge that pro-inflammatory cytokines, such as IL-1β and TNF-α, activate the MAPK pathway, thereby promoting inflammation and metabolic dysfunction [39,40]. Moreover, the pathogenesis of meta-inflammation is closely linked to the well-characterized phenomenon of elevated IL-6 and its receptor expression in the adipose tissue during obesity [41]. TNF-α has also been shown to suppress the cGMP signalling pathway via activation of NF-κB and JNK, impairing adipocyte differentiation and thermogenesis [42]. The enrichment of these pathways in our transcriptomic data not only corroborates the central role of inflammation in obesity but also pinpoints specific signalling cascades that could be targeted to mitigate adipose tissue dysfunction.

Syndecans (SDCs) are a family of type-I transmembrane glycoproteins that function as heparan sulphate proteoglycans, with SDC4 being widely expressed in the body [43] Crocco et al. reported that genetic variants in SDC4 are associated with components of metabolic syndrome in humans, and that Sdc4 knockout in mice affects fat accumulation and insulin sensitivity [44]. Another recent study showed that Sdc4 expression is increased in adipose tissue of obese mice, and Sdc4 protein shedding can inhibit lipolysis, leading to obesity [45]. Clinically, the metabolic relevance of SDC4 is also supported by findings that plasma SDC4 levels in individuals with obesity decreased significantly one year after undergoing bariatric surgery [46]. Consistent with these findings, we found significantly higher SDC4 expression in obese patients’ adipose tissue compared to normal individuals. Furthermore, this upregulated expression was found to be positively correlated with body weight. Considering that bariatric surgery can substantially reduce body fat mass [47], we propose that this may be the underlying reason for the postoperative decrease in plasma SDC4 levels. Further investigation is warranted to quantify SDC4 levels in the serum of patients with obesity and metabolic syndrome, thereby clarifying its potential as a biomarker. Moreover, SDC4 upregulation could be a consequence of obesity-related inflammation or adipocyte hypertrophy. Taken together, these results underscore the role of SDC4 across a spectrum of metabolic pathologies. Consequently, SDC4 emerges as a highly promising target with dual utility: a focal point for the development of novel therapeutic interventions.

Despite these significant findings, our study has several limitations that must be acknowledged. First, the sample size of the sequencing results in this study is relatively small, which may have missed some actual correlations. The results may not be repeatable in another sample, and they may only be applicable to the specific population in this study. In the future, verification is needed in a larger sample. In addition, participants were recruited without strict matching for information such as age or sex, which may have led to instability of the analyses. Second, while our bioinformatics predictions are robust, they require extensive experimental validation. Our study lacks functional assays (e.g. gain- or loss-of-function experiments *in vitro* or *in vivo*) to confirm the causal roles of the identified lncRNAs, miRNAs, and SDC4 in the observed phenotypes. Third, potential batch effects between sample processing and sequencing runs could introduce technical variability, which may impact the reproducibility of our findings. Fourthly, this method may have certain flaws, particularly in the selection of thresholds and databases. This could result in the omission of some crucial lncRNAs, miRNAs, and mRNAs. The selection of the ‘top 300 pairs’ of lncRNAs – miRNAs for network construction may have missed some key interactions. The screening method of DEmiRNA differs from that of lncRNA and mRNA. This may lead to meaningless results, and further molecular experiments are needed for verification in the future. Fifth, for the validation of TNFAIP3, ITPKC, EREG, CYP1A2, and SDC4 in clinical samples, no significant differences were observed for TNFAIP3, ITPKC, EREG, and CYP1A2. This might be related to the relatively small sample size of the normal group. Further verification is recommended in the future, or the functions of each gene can be verified through molecular experiments.

## Conclusion

In summary, this study provides a comprehensive view of the transcriptomic alterations in the VAT of obese individuals. By constructing a ceRNA regulatory network, we have identified novel lncRNA-miRNA-mRNA interactions, highlighting MEG3, hsa-miR-7974, and SDC4 as central nodes in the molecular circuitry of obesity. Our findings offer new perspectives on the pathogenesis of obesity, linking non-coding RNA networks to key inflammatory and metabolic pathways. Despite the limitations of this study, the results provide a candidate for the development of novel therapeutic targets for obesity.

## Supplementary Material

Supplementary Material.docx

## Data Availability

The findings of this study can be openly accessed through the GEO database, which can be found at http://www.ncbi.nlm.nih.gov/geo, under the series accession number GSE235696.
