# Supplementary material for: Identification of SDC4 as a potential target for obesity via integrated analysis of the lncRNA-miRNA-mRNA network in visceral adipose tissue
Source: Adipocyte. 2025 Nov 6;14(1):2583542. doi: 10.1080/21623945.2025.2583542 (PMC12599498; doi:10.1080/21623945.2025.2583542)
Supplement: Supplementary Material.docx [file KADI_A_2583542_SM9207.docx]

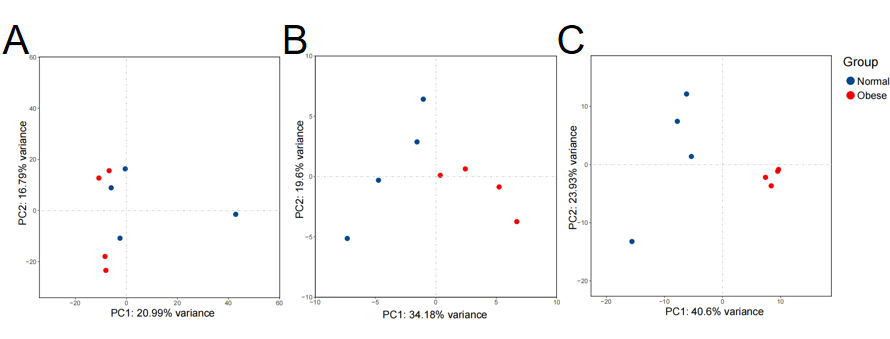


Supplementary figure 1. Principal component analysis (PCA) of sequencing data: A. PCA of LncRNA sequencing results. B. PCA of miRNA sequencing results. C. PCA of mRNA sequencing results.

Supplementary
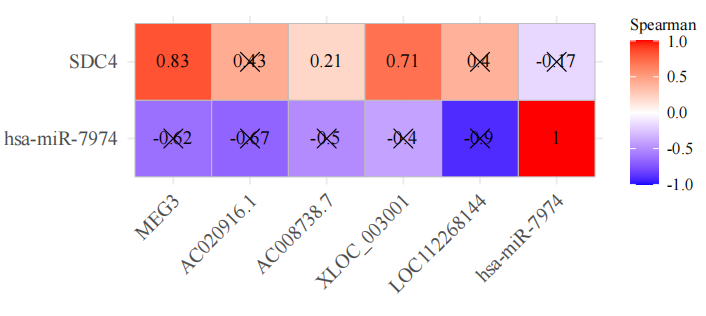
 figure 2. Within the sequencing results, the correlation between MEG3, AC020916.1, AC008738.7, XLOC_003001, LOC112268144, hsa-miR-7974 and SDC4 was detected by Spearman method. Red indicates positive correlation and blue indicates negative correlation.

Supplementary table 1. The top five upregulated and downregulated differentially expressed LncRNAs based on the fold change ranking

| **LncRNA** | **Regulation** | **Log_2_(Fold Change)** | **qValue** |
| --- | --- | --- | --- |
| OVCH1-AS1 | up | 22.0 | q < 0.001 |
| LINC00472 | up | 21.1 | q < 0.001 |
| MEG3 | up | 20.9 | q < 0.001 |
| AC008738.7 | up | 20.7 | q < 0.001 |
| GAS5 | up | 20.2 | q < 0.001 |
| FGD5-AS1 | down | -22.0 | q < 0.001 |
| TTTY10 | down | -21.4 | q < 0.001 |
| TTTY14 | down | -21.2 | q < 0.001 |
| LINC00278 | down | -21.1 | q < 0.001 |
| AC232271.1 | down | -6.6 | p = 0.002 |

Supplementary table 2. The top five upregulated and downregulated differentially expressed miRNAs based on the fold change ranking

| **miRNA** | **Regulation** | **Log_2_(Fold Change)** | **pValue** |
| --- | --- | --- | --- |
| hsa-miR-375-3p | up | 3.8 | p = 0.001 |
| hsa-miR-6843-3p | up | 3.6 | p = 0.049 |
| hsa-miR-6838-5p | up | 3.1 | p = 0.022 |
| hsa-miR-203b-3p | up | 2.2 | p = 0.025 |
| hsa-miR-12130 | up | 2.1 | p = 0.016 |
| hsa-miR-4707-3p | down | -5.1 | p < 0.001 |
| hsa-miR-1973 | down | -4.1 | p = 0.004 |
| hsa-miR-7974 | down | -3.1 | p = 0.001 |
| hsa-miR-1972 | down | -3.0 | p = 0.025 |
| hsa-miR-548ad-5p | down | -2.9 | p = 0.029 |

Supplementary table 3. The top five upregulated and downregulated differentially expressed miRNAs based on the fold change ranking

| **mRNA** | **Regulation** | **Log_2_(Fold Change)** | **qValue** |
| --- | --- | --- | --- |
| PGC | up | 7.8 | q < 0.001 |
| SEZ6 | up | 5.3 | q = 0.017 |
| KISS1R | up | 5.2 | q = 0.036 |
| CSF3 | up | 5.1 | q < 0.001 |
| SELE | up | 4.9 | q < 0.001 |
| UTY | down | -9.9 | q = 0.029 |
| DDX3Y | down | -9.8 | q = 0.005 |
| RPS4Y1 | down | -8.8 | q = 0.003 |
| ADH4 | down | -2.8 | q = 0.040 |
| CYP1A2 | down | -2.7 | q = 0.033 |

Supplementary table 4. All differential expression mRNAs and KEGG pathway enrichment analysis

| Ontology | ID | Description | p.adjust | Gene ID |
| --- | --- | --- | --- | --- |
| BP | GO:0071347 | cellular response to interleukin-1 | <0.001 | CCL7/CCL3L3/CCL3/EGR1/CCL2/  CL4/CCL8/MYC/IL1R2/IL1RN/  ZC3H12A/IL1B/HAS2/KLF2/HES1 |
| BP | GO:0070555 | response to interleukin-1 | <0.001 | SELE/CCL7/CCL3L3/CCL3/EGR1/CCL2/CCL4/CCL8/MYC/IL1R2/  IL1RN/ZC3H12A/IL1B/HAS2  /KLF2/HES1 |
| BP | GO:0050900 | leukocyte migration | <0.001 | SELE/CCL7/CCL3L3/CCL3/CCL2/GPR15/CXCR1/SERPINE1/TREM1/CCL4/CH25H/CCL8/CXCR2/  BDKRB1/DUSP1/IL10/CXCL3/  TNFAIP6/THBS1/GPR183/IL1B/  C5AR1/ICAM1/ADORA1 |
| BP | GO:0060326 | cell chemotaxis | <0.001 | CCL7/CCL3L3/EGR3/NR4A1/  CCL3/CCL2/CXCR1/SERPINE1/  TREM1/CCL4/CH25H/CCL8/  CXCR2/DUSP1/IL10/HBEGF  /CXCL3/TNFAIP6/THBS1/GPR183/IL1B/C5AR1 |
| BP | GO:0002237 | response to molecule of bacterial origin | <0.001 | CSF3/SELE/IL24/PTGS2/NR4A1/  CCL3/CCL2/SERPINE1/FOS/  ZFP36/BDKRB1/ARID5A/IL10/  CXCL3/THBD/ZC3H12A/TRIB1  /IL1B/C5AR1/JUND/TNFAIP3/  CD55/SLC11A1 |
| CC | GO:0030667 | secretory granule membrane | <0.001 | OLR1/VAMP7/CXCR1/FCGR3B/  CXCR2/ADGRE3/MMP25/PLAUR/  SLC2A3/MGAM/C5AR1/SIRPB1/  ITGAX/CD55/SLC11A1 |
| CC | GO:0090575 | RNA polymerase II transcription regulator complex | =0.001 | FOSL1/ATF3/FOS/JUNB/MYC/  MAFF/JUN/FOSL2/CREM/JUND/  NFIL3 |
| CC | GO:0005667 | transcription regulator complex | =0.001 | NR4A3/FOSL1/NR4A2/NR4A1/  ATF3/FOS/JUNB/ARID5A/MYC/MAFF/JUN/FOSL2/KLF4/CREM  /JUND/NFIL3 |
| CC | GO:0101003 | ficolin-1-rich granule membrane | =0.001 | ADGRE3/SLC2A3/MGAM/ITGAX/CD55/SLC11A1 |
| CC | GO:0030669 | clathrin-coated endocytic vesicle membrane | =0.003 | EREG/AREG/VAMP7/HBEGF  /LDLR/IL7R |
| MF | GO:0005125 | cytokine activity | <0.001 | CSF3/IL24/CCL7/CCL3L3/CCL3/  AREG/CCL2/CCL4/IL20/OSM/  CCL8/IL10/TNFSF9/LIF/CXCL3/  CLCF1/IL1RN/IL1B/NAMPT |
| MF | GO:0005126 | cytokine receptor binding | <0.001 | CSF3/CCL7/CCL3L3/CCL3/CCL2/CCL4/IL20/OSM/CCL8/IL10/  TNFSF9/LIF/CXCL3/CLCF1/  BDNF/IL1RN/TRAF4/IL1B/  BAMBI/ITGA5 |
| MF | GO:0048018 | receptor ligand activity | <0.001 | CSF3/IL24/CCL7/CCL3L3/CCL3/  EREG/AREG/CCL2/CHGB/  SEMA4A/CCL4/IL20/OSM/CCL8/  IL10/TNFSF9/HBEGF/LIF/CXCL3/CLCF1/BDNF/IL1RN/IL1B/STC1/NAMPT |
| MF | GO:0030546 | signaling receptor activator activity | <0.001 | CSF3/IL24/CCL7/CCL3L3/CCL3/  EREG/AREG/CCL2/CHGB/  SEMA4A/CCL4/IL20/OSM/CCL8/  IL10/TNFSF9/HBEGF/LIF/CXCL3/CLCF1/BDNF/IL1RN/IL1B/STC1/NAMPT |
| MF | GO:0001228 | DNA-binding transcription activator activity, RNA polymerase II-specific | <0.001 | NR4A3/EGR2/FOSB/EGR3/FOSL1/NR4A2/NR4A1/ATF3/EGR1/  CSRNP1/FOS/JUNB/MYC/MAFF/  JUN/FOSL2/KLF4/KLF10/JUND |
| KEGG | hsa04060 | Cytokine-cytokine receptor interaction | <0.001 | CSF3/IL24/CCL7/CCL4L2/CCL3L3/CCL3/CCL2/CXCR1/CCL4/IL20  /IL3RA/OSM/CCL8/CXCR2/TNFRSF12A/IL10/TNFSF9/IL1R2/LIF/  CXCL3/TNFRSF10D/CLCF1/  IL1RN/TNFRSF10C/IL7R/IL1B/  IL1RL1 |
| KEGG | hsa04061 | Viral protein interaction with cytokine and cytokine receptor | <0.001 | IL24/CCL7/CCL4L2/CCL3L3/  CCL3/CCL2/CXCR1/CCL4/IL20/  CCL8/CXCR2/IL10/CXCL3/  TNFRSF10D/TNFRSF10C |
| KEGG | hsa04668 | TNF signaling pathway | <0.001 | SELE/PTGS2/SOCS3/CCL2/  FOS/JUNB/LIF/CXCL3/JUN/BCL3/IL1B/TNFAIP3/MAP3K8/BIRC3/  ICAM1 |
| KEGG | hsa04657 | IL-17 signaling pathway | <0.001 | CSF3/CCL7/FOSB/FOSL1/PTGS2/CCL2/FOS/CXCL3/JUN/TRAF4/  IL1B/JUND/TNFAIP3 |
| KEGG | hsa04630 | JAK-STAT signaling pathway | <0.001 | CSF3/IL24/SOCS3/IL20/CDKN1A/IL3RA/OSM/MYC/IL10/LIF/IL7R/CISH/MCL1 |
| KEGG | hsa04380 | Osteoclast differentiation | <0.001 | FOSB/FOSL1/SOCS3/FOS/  FCGR3B/JUNB/JUN/FOSL2/IL1B/SIRPB1/JUND |
| KEGG | hsa05418 | Fluid shear stress and atherosclerosis | <0.001 | SELE/CCL2/FOS/DUSP1/IL1R2/  THBD/JUN/IL1B/SDC4/KLF2/  ICAM1 |
| KEGG | hsa04010 | MAPK signaling pathway | <0.001 | NR4A1/EREG/AREG/FOS/DUSP2/DUSP5/MYC/DUSP1/GADD45B/  BDNF/JUN/DUSP8/FGFR3/IL1B/  JUND/MAP3K8 |
| KEGG | hsa05144 | Malaria | <0.001 | CSF3/SELE/CCL2/IL10/THBS1/  IL1B/ICAM1 |
| KEGG | hsa04064 | NF-kappa B signaling pathway | <0.001 | CCL4L2/PTGS2/CCL4/GADD45B/CXCL3/IL1B/TNFAIP3/BIRC3/  ICAM1 |
